# Correction to “Moderate Drought Constrains Crop Growth Without Altering Soil Organic Carbon Dynamics in Perennial Cup‐Plant and Silage Maize”

**DOI:** 10.1111/gcbb.70013

**Published:** 2024-11-29

Abdalla, K., Uther, H., Kurbel, V. B., Wild, A. J., Lauerer, M., Pausch, J. 2024. Moderate Drought Constrains Crop Growth Without Altering Soil Organic Carbon Dynamics in Perennial Cup‐Plant and Silage Maize. Global Change Biology Bioenergy 16:e70007, https://doi.org/10.1111/gcbb.70007

In the article by Abdalla et al. (2024), we found an error in the unit of soil organic carbon (SOC) stocks in Figure 3d–f. Specifically, the original unit was given as **g** C m^−2^, but it should be **kg** C m^−2^. In addition, it was not clearly stated in the figure legend that these data represent an average of 9 soil depths of 10 cm each (0–90 cm profile).

**FIGURE 3:**
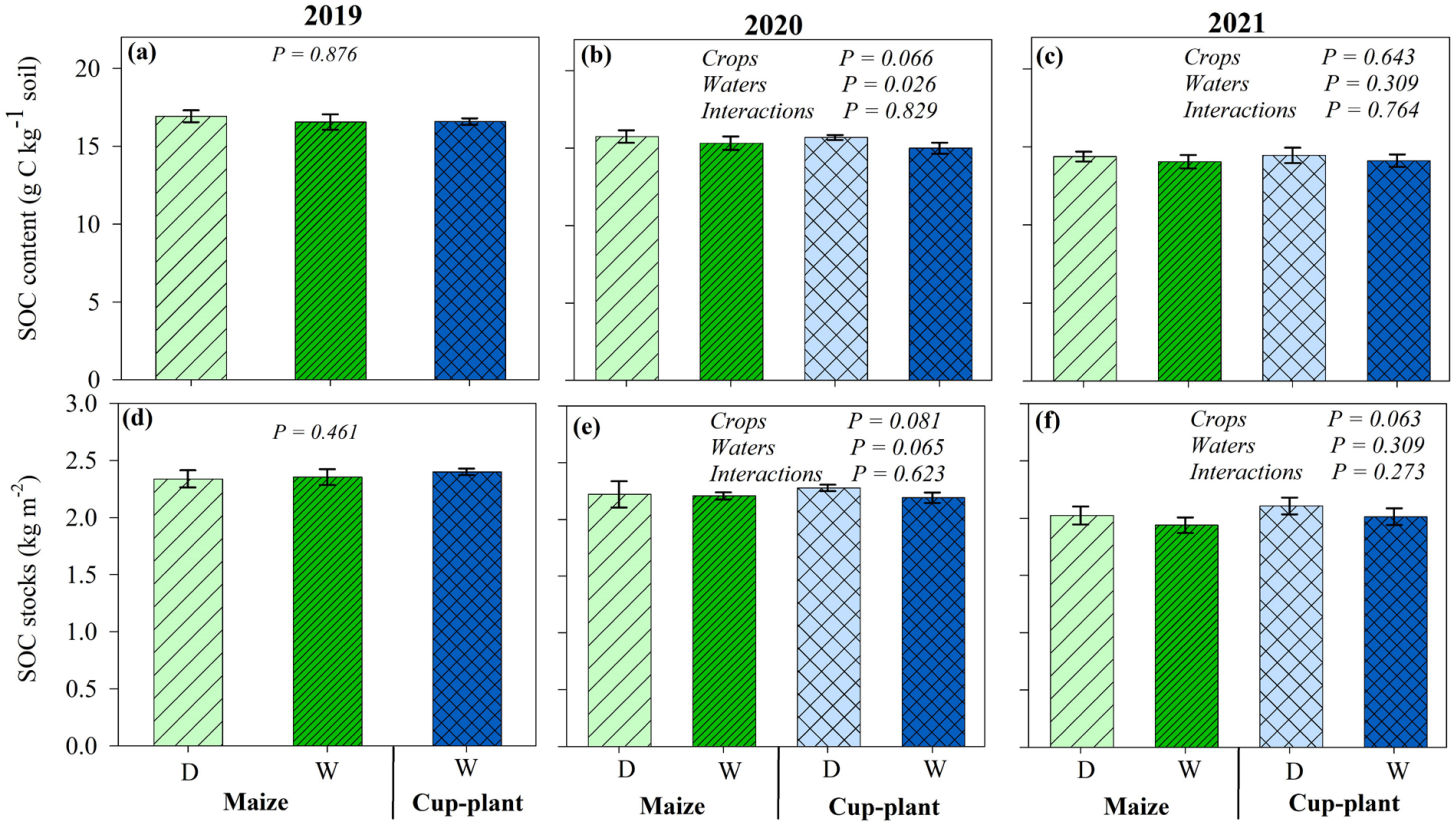
Mean ± SE of the soil organic carbon (SOC) content (a–c) and stocks (d–f) of the soil cultivated with maize and cup‐plant under moderate drought (D) and well‐watered (W) conditions over the growing seasons of 2019, 2020 and 2021. The data represent an average of 9 soil layers of 10 cm each (0–90 cm depth). Means followed by different letters within 1 year are significantly different at *p* ≤ 0.05; (*n* = 30).

Another minor error in the unit of Figure 5b, where the unit of the microbial biomass nitrogen was given in mg **C** kg^−1^ soil, which should be mg **N** kg^−1^ soil.

We apologise for any inconvenience this error may cause.

